# Bone Marrow Senescence and the Microenvironment of Hematological Malignancies

**DOI:** 10.3389/fonc.2020.00230

**Published:** 2020-02-25

**Authors:** Charlotte Hellmich, Jamie A. Moore, Kristian M. Bowles, Stuart A. Rushworth

**Affiliations:** ^1^Norwich Medical School, University of East Anglia, Norwich, United Kingdom; ^2^Department of Haematology, Norfolk and Norwich University Hospitals NHS Trust, Norwich, United Kingdom

**Keywords:** senescence, cancer microenvironment, leukemia, p16INK4a, SASP

## Abstract

Senescence is the irreversible arrest of cell proliferation that has now been shown to play an important role in both health and disease. With increasing age senescent cells accumulate throughout the body, including the bone marrow and this has been associated with a number of age-related pathologies including malignancies. It has been shown that the senescence associated secretory phenotype (SASP) creates a pro-tumoural environment that supports proliferation and survival of malignant cells. Understanding the role of senescent cells in tumor development better may help us to identify new treatment targets to impair tumor survival and reduce treatment resistance. In this review, we will specifically discuss the role of senescence in the aging bone marrow (BM) microenvironment. Many BM disorders are age-related diseases and highly dependent on the BM microenvironment. Despite advances in drug development the prognosis particularly for older patients remains poor and new treatment approaches are needed to improve outcomes for patients. In this review, we will focus on the relationship of senescence and hematological malignancies, how senescence promotes cancer development and how malignant cells induce senescence.

## Introduction

Bone marrow disorders, including myeloproliferative neoplasm (MPN), myelodysplastic syndrome (MDS), leukemia, and multiple myeloma are largely diseases of the elderly and as our population ages their incidence will likely continue to increase and with it, disease associated mortality. Acute myeloid leukemia (AML), alone accounted for 85,000 deaths globally in 2016 and multiple myeloma caused 98,000 deaths (1). As an example, AML has a peak incidence between the ages of 80 and 85 years (2); its prognosis remains especially poor for many of our older patients who cannot tolerate the intensive chemotherapies available, with a 5-year survival rate of only 5% in patients over the age of 65. Even younger patients, who achieve remission, frequently relapse from minimal residual diseased cells sequestered in the BM. Whilst, the outcomes for patients with multiple myeloma have improved with new therapies including lenalidomide, bortezomib, autologous stem cell transplantation and most recently daratumumab, it remains a largely incurable disease and despite achieving periods of remission most patients will eventually relapse (3). Hematopoietic malignancies including AML multiple myeloma, MPNs and MDS are highly dependent on the BM microenvironment for survival (3–7).

The BM is the primary hematopoietic organ in adults. It comprises of blood vessels, nerve tissue and a heterogeneous population of cells that are either directly involved in the generation of blood cells or support the hematopoietic function of the tissue (8, 9). Together, all the components of the BM tightly regulate normal hematopoiesis to ensure adequate production of mature blood cells. In blood cancers, however, this process is disrupted resulting in cytopenias and immunosuppression. In AML, this is thought to be instigated by leukemic cells directly blocking differentiation of normal hematopoietic stem cells (HSC) (10), as well as the manipulation of other BM-derived cells (including macrophages, endothelial cells, fibroblasts, and adipocytes) (4, 6, 11, 12). The hallmarks of Myeloma include anemia, which is partly due to bone marrow failure, and lytic bone disease caused by an imbalance of osteoclast and osteoblast activity, which is mediated by the malignant plasma cells (13–15). These are only two examples of how malignant cells alter their microenvironment to promote tumor survival and proliferation (4–6, 16). Given the impact tumor cells have on their surrounding cells, it follows that this interaction between malignant cells and the BM microenvironment could be utilized to explore new treatment approaches that are clearly needed for these lethal diseases.

## Senescence

Cellular senescence is the irreversible arrest of cell proliferation (17). It is associated with the secretion of numerous pro-inflammatory cytokines, chemokines, proteases, and growth factors, known as the senescence-associated secretory phenotype (SASP). It occurs as a response to cellular damage and is thought to have evolved to both suppress development of cancer and to promote tissue repair and wound healing (18). In addition to cellular damage a number of external triggers, including oxidative stress, radiation, and cytotoxic chemotherapy can cause DNA damage and as a result induce cellular senescence. It is mediated by the cyclin-dependent kinase inhibitors p16 and p21, which mediate cell cycle arrest through the retinoblastoma and p53 tumor suppressor pathways, respectively (19). In the short term the SASP plays an important role in recruiting immune cells to sites of cellular damage in order to promote tissue repair, limit tissue fibrosis and clear senescent cells. However, it appears that this process becomes less effective with age and senescent cells gradually accumulate. Overall the senescent response becomes maladaptive and there is now increasing evidence that it contributes to a number of age-related phenotypes and pathologies. When it persists over time the SASP has paradoxically been shown to disrupt a number of cellular and tissue functions to create a pro-tumoral and chemotherapy-resistant environment (19, 20).

Activation of tumor suppressor pathways prevents cell cycle progression and can cause either activation of downstream pathways that eventually result in apoptosis or pathways that ultimately induce a senescent phenotype. The exact mechanisms by which the fate of the cell is determined is not yet fully understood but a number of factors appear to contribute. These includes the type of cell, the degree of stress which for example includes the dose of chemotherapy and the balance of pro-senescent and pro-apoptotic signals which are at least partly determined by the cell's microenvironment (21–23). It appears that once the cell commits to the senescent phenotype it is protected from pro-apoptotic stimuli (24).

A number of different models to study senescence *in vivo* now exist. These include models that allow identification of senescent cells *ex vivo* using fluorescent tags (25), detection of senescent populations *in vivo* (26, 27), and selective elimination of senescent cells (25). In the p16-3MR model, developed by the Campisi group, these are all combined and the p16 promotor drives expression of renilla luciferase, red fluorescent protein (RFP) and HSV thymidine kinase. This allows *in vivo* imaging of senescent cells using luminescence, isolation of senescent cells *in vitro* and selective depletion of senescent cells using the pro-drug ganciclovir (18). The limitation of the p16-3MR model is the low signal of both the renilla luciferase and the RFP. It is not possible to detect the renilla signal within deep tissues or the bone marrow *in vivo*. Moreover, the p16 driven RFP signal is weak and therefore difficult to detect *ex vivo*. To address this, the Sharpless group developed a model using the ultrabright fluorochrome tandem dimer Tomato (p16-tdTom), which allows for better analysis and isolation of senescent cells (28). However, unlike the p16-3MR model, this model has no way of depleting senescent cells, an aspect that is important to consider when studying the role of senescence in aging and age-related diseases. Thus, the ideal model to study senescence *in vivo* would incorporate the brightness of the p16-tdTom with the depletion aspect of the p16-3MR model.

## Senescence in the Aging Bone Marrow

The bone marrow is the primary site of hematopoiesis in adults. HSCs proliferate and differentiate to produce mature myeloid, lymphoid and erythroid cells and platelets. Supporting cells, including endothelial cells, fibroblasts, osteoblasts, and adipocytes help to regulate this process and ensure a balanced production of mature blood cells. With age the bone marrow structure changes significantly, as the cellular component is gradually replaced by adipose tissue (29). The proportion of highly hematopoietically active red marrow gradually falls and there as an increase in fatty non-hematopoietic yellow marrow (30, 31). Furthermore, HSCs from aged mice have altered gene expression with an upregulation of genes involved in inflammatory and stress responses (32) as well as reduced self-renewal and long-term repopulation ability with skewed differentiation toward the myeloid lineage (33, 34). Thus, whilst some normal hemtopoiesis continues, with age HSCs gradually decline in function, resulting in dysregulation of normal hematopoiesis. This directly alters the BM microenvironment and likely contributes to the pathogenesis of the many age-related bone marrow disorders, including AML, chronic myeloid leukemia, chronic lymphocytic leukemia and myleloma. In addition, these changes in the HSC pool impact on the immune system and immunosurveillance, a process known as immunosenescence (35, 36). This not only affects the bone marrow microenvironment but has much broader health implications for our aging populations as it contributes to other age-related disease, such as infectious diseases, autoimmune diseases and solid tumors.

## Clonal Hematopoiesis

Increasing age is associated with an accumulation of mutations and the nature of the mutation determines the cell's fate. The bone marrow is a site of very high cell turnover with trillions of cells being produced daily through clonal expansion of HSCs and progenitor cells (37). Mutations may give a selective survival and proliferative advantage and if they arise within the HSC or early progenitor cells, they will be passed down to all daughter cells and as a result are detectable in cells circulating in the peripheral blood. Clonally expanding cell populations can be detected in patients with pre-malignant conditions, such as monoclonal gammopathy of unknown significance (MGUS), in which an initiating event results in clonal proliferation of plasma cells but this only progresses to multiple myeloma if further mutations are acquired (38). Clonal hematopoiesis can be triggered by skewed X chromosome inactivation as well as somatic mutations, including most commonly in the DNM3TA, TET2, and ASXL1 genes (39, 40). These mutations are commonly associated with myeloproliferative disorders, MDS and AML but clonal hematopoiesis associated with these mutations has also been linked to other age-related pathologies including cardiovascular disease even in the absence of any hematological disorder (41). The exact mechanism for this remains to be fully explored but this may be related to altered macrophage function resulting in impaired immunosurveillance which could lead to reduced clearance of senescent cells in these tissues. It is possible that age-related changes within the BM microenvironment, including accumulation of senescent cells, pro-inflammatory changes and the secretion of the SASP, favor clonal expansions as mutated cells adapt to survive and proliferate in the increasingly adverse environment, whilst normal hematopoiesis is impaired in these conditions.

## Chemotherapy Induced Senescence

**Figure 1:**
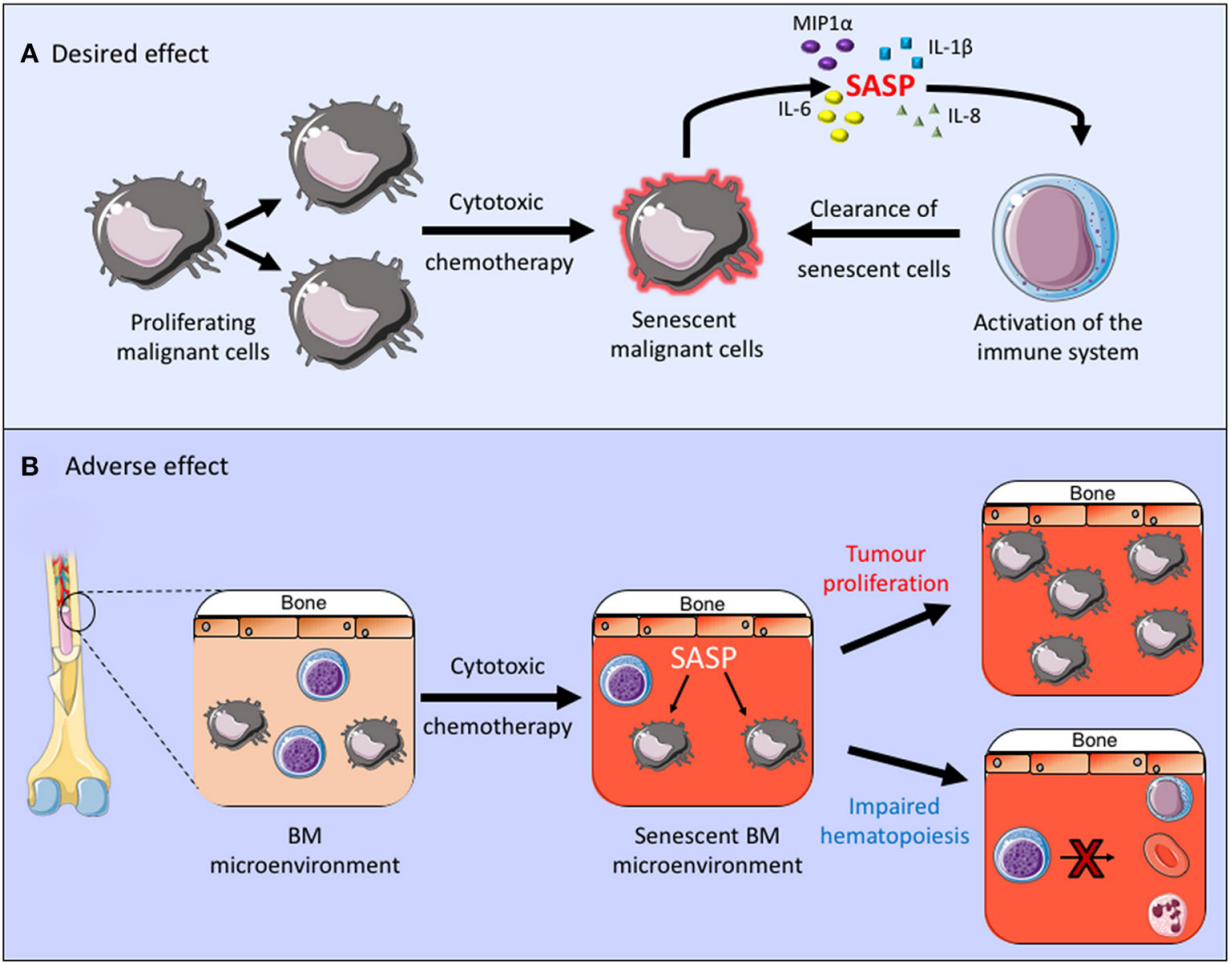
**(A,B)** The desired and adverse effect of cytotoxic chemotherapy. By inducing senescence in the malignant cell these treatments inhibit cell proliferation and promote clearance of senescent cells through activation of the immune system. Conversely when a senescent phenotype occurs within the tumor microenvironment this can promote chronic inflammation, tumor growth and it can impair normal bone marrow function.

Cytotoxic chemotherapy drugs are used widely in the management of both solid and hematological malignancies. AML is a good example in which cytotoxic drugs are frequently used and senescence is known to contribute to the disease pathogenesis. Although new drugs are being introduced into treatment regimens for AML, including targeted treatments, such as FLT3 inhibitors and immunotherapy agents, the backbone of AML treatment has been the same for many years and consists of daunorubicin and cytarabine (42). Daunorubicin is an anthracycline cytotoxic antibiotic which intercalates with DNA and inhibits topoisomerase II function, resulting in a DNA damage response and its cytotoxic effect. Cytarabine is an antimetabolite which inhibits DNA synthesis and its action is specific to the S-phase of the cell cycle. Together these cytotoxic agents alter the cellular state which can, not only lead directly to cell death but it can also induce a senescent state. This is partly a desired effect in the treatment of malignancies as it inhibits the proliferation of malignant cells and can also stimulate immunosurveillance and therefore clearance of malignant cells (43, 44). In fact, the method of action of retinoic acid and arsenic trioxide, the only curative targeted cancer therapy used in the treatment of acute promyelocytic leukemia, is to induce senescence in the leukemic cells through activation of the p53 pathway (45) (Figure 1A). Other cytotoxic chemotherapy agents, however, are non-specific and they can therefore also induce senescence in healthy tissues (46), thus impacting on the tumor microenvironment and other healthy organs and tissues. A number of chemotherapy agents with varying modes of action, including anthracyclines have been shown induce senescence in non-cancerous tissue, resulting in the generation of the SASP, which creates a chronic inflammatory state that is thought to contribute to the side effects associated with chemotherapy treatments (20, 47). Furthermore, clearance of senescent cells within the tumor microenvironment was shown to reduce recurrence and metastases of solid tumor (46). Thus, in the tumor microenvironment senescent cells not only affect the normal function of healthy tissues but they can also promote tumor survival (Figure 1B).

## Senescence in the Tumor Microenvironment

**Figure 2:**
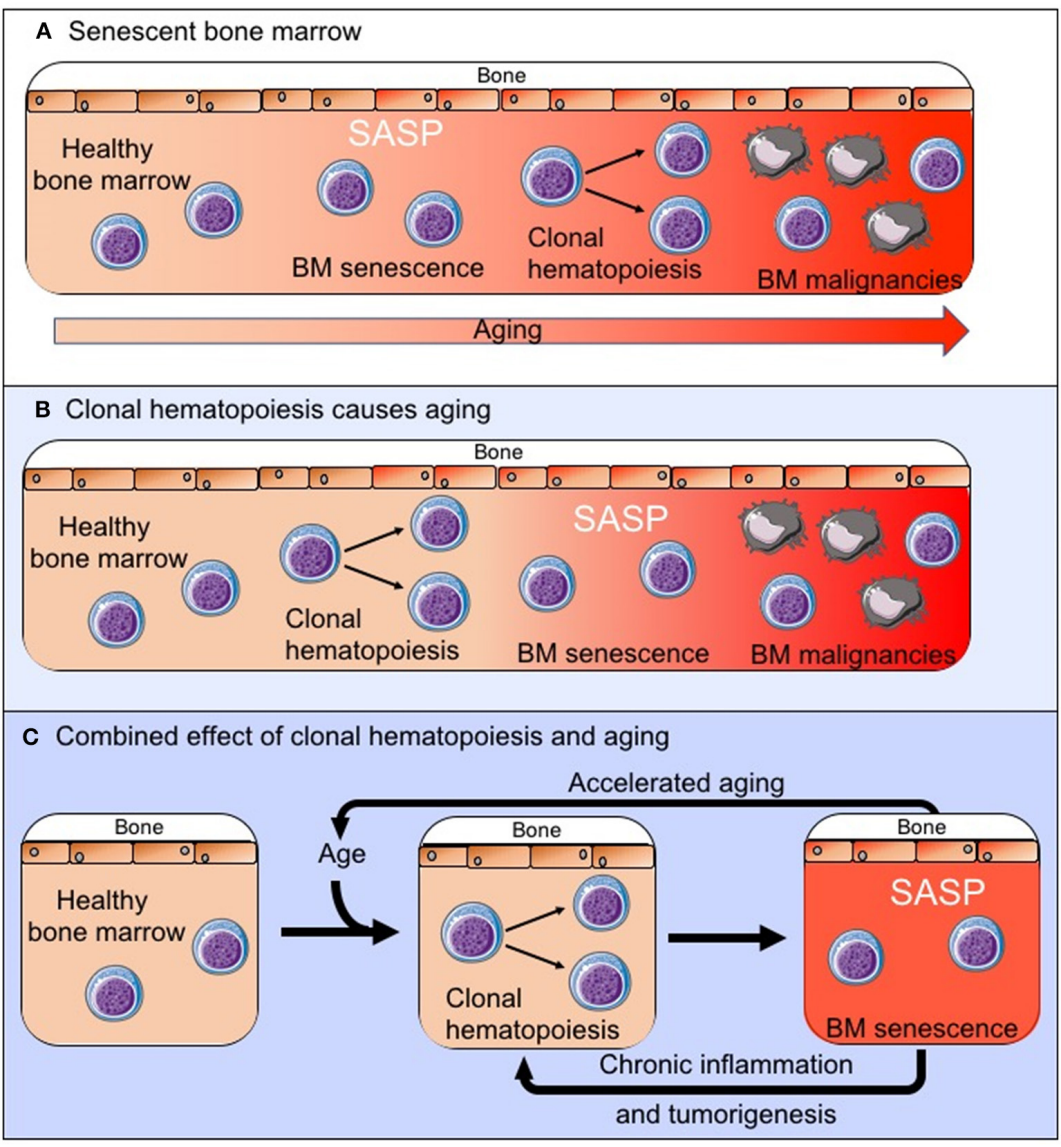
The exact relationship between the senescent phenotype in the BM microenvironment and tumorigenesis remains to be established. We propose three possible pathways by which the senescent pro-tumoral BM microenvironment is created: **(A)** The senescent process is driven by normal aging and external factors and this creates an environment in which clonal hematopoiesis and tumorigenesis is favored. **(B)** The clonally expanding cell populations are the primary drivers of senescence in the BM microenvironment. **(C)** These two processes occur in parallel, impact on each other to varying degrees and together drive the observed changes in the BM.

There is increasing evidence that the tumor microenvironment is crucial for tumor development, survival and drug resistance (3–7, 16). It should therefore be considered that age related changes within the BM microenvironment contribute to the development of hematological malignancies. These changes can also affect disease progression and response to treatment, and this may contribute, for example, to poorer outcomes observed in older patients with AML, which are not sufficiently explained by the differences in adverse prognostic features (48). AML cells were shown to induce a senescent phenotype in BM stromal cells (BMSCs) resulting in the secretion of a SASP which supports the survival and proliferation of leukemic blasts. *In vivo* experiments, using the p16-3MR model of senescence, showed that leukemic blast derived superoxide induces p16INK4A driven senescence in BMSCs (CD45-, CD105+, CD140a+, CD31– and ter119–) and that deletion of these senescent BMSCs slows tumor progression and prolongs animal survival (49). As another example multiple myeloma cells have been shown to induce a SASP (50) and senescence in mesenchymal stem cells which creates an environment that supports myeloma cells growth (51, 52), although the exact relationship between the myeloma cells and the senescent mesenchymal stem cells remains to be explored further (53). However, as with a number of solid tumors it is becoming increasingly clear that a senescent microenvironment favors survival of malignant cells in the bone marrow. It remains yet to be determined whether an existing senescent environment, as is observed with increasing age (Figure 2A), drives the development of these malignancies or whether in fact the expansion of clonal cell populations within the bone marrow microenvironment drives the senescent process (Figure 2B), accelerates aging and impairs immunosurveillance and clearance of both senescent cells and pre-malignant cells. It is also possible that these two processes together create the senescent BM microenvironment that is observed both with increasing age and in BM malignancies (Figure 2C). However, as there is increasing evidence that senescence in the bone marrow microenvironment forms a fundamental part of the malignant phenotype, this raises the question whether targeting the “benign” senescent cells in the BM microenvironment could disrupt the supportive nature of the tumor microenvironment and as a result impair tumor survival. Thus, providing a potential new treatment target.

## Anti-Senolytics as Potential Therapy for AML

The p16-3MR mouse model of senescence was used to show that AML induces senescence in its microenvironment and that this occurs in the absence of any treatment with chemotherapy (49). Furthermore, it is known that many of the cytotoxic treatments used to treat AML can induce senescence in healthy tissues including the bone marrow microenvironment. Thus, both AML and its treatment will contribute to a senescent phenotype in the bone marrow environment. This has been shown to promote tumor survival but at the same time impair normal hematopoiesis (19, 35, 46, 49), which may not only contribute to treatment resistance and relapse of disease but also to immediate and long-term side effects associated with chemotherapy treatments. It therefore follows that it would be beneficial to eliminate senescent cells in the BM microenvironment. A number of senolytic agents which can selectively eliminate senescent cells have been identified (24, 54–56). The anti-apoptotic proteins BCL-2 and BCL-XL have been implicated in protecting senescent cells from pro-apoptotic signals and a number of inhibitors of the BCL-2 family have been developed. Of these venetoclax, a selective BCL-2 inhibitor (57) has been licensed for clinical use and is currently used in the treatment of chronic lymphocytic leukemia and AML. It is an example of how targeted treatments can be effective in treating older patients whilst causing less toxicity than other existing cytotoxic treatment regimens (58, 59). However, despite its action as a BCL-2 inhibitor, venetoclax does not have any significant senolytic activity (24, 54). Navitoclax, on the other hand, inhibits both BCL-2 as well as BCL-XL and has been shown to selectively induce apoptosis in senescent cells in the p16-3MR mouse model, including senescent HSCs with subsequent recovery of the hematopoietic system (54). However, given the nature of HSC it could be debated whether it is possible for them to become senescent, thus, the observed effect could be a result of depletion of other senescent cells of the bone marrow microenvironment. Alternatively, high levels of expression of BCL-XL and BCL-2 may not be unique to senescent cells, and therefore navitoclax may not be specific to senescent cells in this context; this could have implications for its clinical use and would require further investigation. It would be of interest to further investigate BCL-XL inhibitors in isolation to determine if BCL-XL inhibition is sufficient to deplete senescent cells or if simultaneous inhibition of both BCL-2 and BCL-XL is required. No work has yet shown whether this would be transferrable to the leukemic microenvironment or other hematological malignancies, however, if senescent cells can be eliminated from the tumor microenvironment this may not only affect tumor survival and chemotherapy resistance but also promote recovery of normal hematopoiesis and immunosurveillance within the bone marrow.

## Conclusion

As our population ages, the burden of age-related diseases will increase, and new treatment approaches are being explored to improve heath-span and reduce long-term disease burdens. For this it is essential to first understand the relationship between aging and disease. Here we have focused on bone marrow malignancies, their dependence on the bone marrow microenvironment and the role of senescence in their development, survival and treatment. Senescence is a complex process involving numerous cellular and molecular pathways that are influenced by internal and external triggers and whilst our understanding of these mechanisms is increasing, many unanswered questions remain. We know that cellular senescence plays a vital role in wound healing, tumor suppression and treatment of malignancies but that it also can have a detrimental effect and contribute to age-related pathologies including hematological malignancies. There is clearly a fine balance between the beneficial and detrimental effects of cellular senescence and understanding this balance will be crucial if we wish to exploit this process in the treatment of cancer and other age-related diseases.

## Author Contributions

CH, JM, KB, and SR wrote the paper. CH and JM made the figures.

### Conflict of Interest

The authors declare that the research was conducted in the absence of any commercial or financial relationships that could be construed as a potential conflict of interest.
